# Beyond the glitter: gold nanoparticles as powerful weapons against multi-drug resistant pathogens

**DOI:** 10.3389/fmolb.2025.1612526

**Published:** 2025-08-11

**Authors:** Hazim O. Khalifa, Hind Alkhoori

**Affiliations:** ^1^ Department of Veterinary Medicine, College of Agriculture and Veterinary Medicine, United Arab Emirates University, Al-Ain, United Arab Emirates; ^2^ UAEU Center for Public Policy and Leadership, United Arab Emirates University, Al Ain, United Arab Emirates

**Keywords:** gold nanoparticles, multidrug-resistant pathogens, antimicrobial resistance, biofilm disruption, reactive oxygen species, antibiotic synergy, fungal infections, gram-negative bacteria

## Abstract

Gold nanoparticles (AuNPs) have emerged as promising antimicrobial agents in the fight against multidrug-resistant (MDR) pathogens. Their distinctive physicochemical properties allow them to target a broad spectrum of MDR microorganisms, including highly virulent strains such as methicillin-resistant *Staphylococcus aureus* (MRSA), *Pseudomonas aeruginosa*, *Escherichia coli*, *Acinetobacter baumannii*, and *Candida albicans*. AuNPs exert potent antimicrobial effects through various mechanisms, including bacterial growth inhibition, biofilm disruption, reactive oxygen species (ROS) generation, and enhancement of conventional antibiotic efficacy. Compared to traditional antimicrobials, these nanoparticles offer key advantages such as low toxicity, high biocompatibility, and a reduced likelihood of promoting bacterial resistance. This review provides a comprehensive analysis of the antimicrobial mechanisms, synergistic interactions with antibiotics, and therapeutic potential of AuNPs. Additionally, it examines recent advancements in their clinical applications, formulation strategies, and safety profiles. Despite encouraging results, challenges persist in optimizing AuNP synthesis, evaluating their long-term effects, and ensuring their large-scale clinical translation. Future research should focus on improving nanoparticle formulations, assessing their *in vivo* efficacy, and conducting extensive clinical trials to confirm their therapeutic viability. Overall, AuNPs represent a promising and multifaceted approach to tackling antimicrobial resistance, offering new avenues for the development of effective treatments against MDR pathogens.

## 1 Introduction

Multidrug resistance (MDR) in pathogens—bacteria, fungi, parasites, and viruses—has become a major global health challenge (Khalifa et al., 2021a). The rapid evolution of MDR hinders the effectiveness of existing drugs, forcing pharmaceutical companies to develop new therapies. Although antimicrobial resistance (AMR) is a natural phenomenon, human activities have significantly accelerated the process, posing a worldwide threat (Murray et al., 2022; Khalifa et al., 2022a). In 2019 alone, AMR directly caused an estimated 1.27 million deaths and contributed to 4.95 million deaths, highlighting the urgency of this issue (Murray et al., 2022). If left unchecked, AMR could lead to 10 million deaths annually by 2050, surpassing major diseases like cancer in mortality rates (Alaoui Mdarhri et al., 2022). The economic consequences are equally severe, potentially pushing 24 million people into extreme poverty (Alaoui Mdarhri et al., 2022). A 2019 report by the U.S. Centers for Disease Control and Prevention (CDC) found that AMR has raised healthcare costs by $20 billion, excluding an estimated annual economic loss of $35 billion (Centers for Disease Control and Prevention, 2019). The report also underscores the human impact, revealing that approximately 23,000 deaths occur each year among two million individuals affected by antimicrobial-resistant infections in the United States. Despite continuous efforts to develop alternative treatments, no current approach can entirely replace traditional antibiotics (Ahmed et al., 2015; Khalifa et al., 2021b). Addressing this crisis requires a comprehensive, multidisciplinary strategy that integrates public health, pharmaceutical science, and biotechnology to develop innovative and effective solutions against antimicrobial resistance (Al-Hakkani et al., 2023; Khalifa et al., 2024b).

Given the declining efficacy of conventional antibiotics, researchers are exploring gold nanoparticles (AuNPs) as a novel antimicrobial agent. Nanotechnology offers a high drug-loading capacity and enhanced tissue penetration, making it a promising alternative (Shamaila et al., 2016). AuNPs exhibit unique properties, including high specificity in targeting microbial cells, low toxicity, and strong biocompatibility (Osonga et al., 2020). Moreover, AuNPs disrupt bacterial structures, enhancing their antimicrobial effectiveness. This was further supported by studies demonstrating other nanoparticles such as silver nanoparticles (AgNPs) which interact with bacterial membranes and generating reactive oxygen species, leading to bacterial cell death (Park et al., 2010). These findings suggest that nanoparticles could potentially overcome MDR by attacking pathogens through multiple mechanisms, reducing the likelihood of resistance development. In recent years, numerous studies have explored antimicrobial strategies to combat bacterial infections. To streamline information for readers, this review provides a comprehensive analysis of the latest advancements in AuNPs against MDR pathogens. We examine their properties, antimicrobial mechanisms, spectrum of activity, targeted delivery systems, resistance challenges, and biocompatibility. Finally, we discuss future directions to enhance AuNP efficacy and broaden their medical applications.

## 2 Gold nanoparticles (AuNPs): properties and synthesis

AuNPs exhibit unique physicochemical properties that make them highly valuable in biomedical and technological applications. In this section, we will explore key characteristics of AuNPs, including size and shape, which significantly influence their optical properties, surface chemistry, and chemical stability. Additionally, we will discuss their catalytic activity and biocompatibility, which play crucial roles in their biomedical use. Furthermore, we will examine various synthesis techniques such as the chemical reduction method, seed-mediated growth method, template-assisted synthesis, and electrochemical synthesis, each of which contributes to tailoring AuNPs for specific applications. Understanding these properties and synthesis methods is essential for optimizing their functionality in medical and industrial fields.

### 2.1 Properties of AuNPs

**FIGURE 1:**
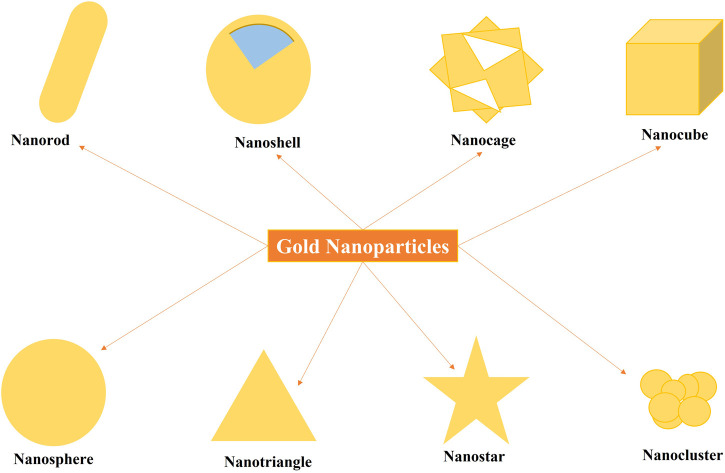
Illustration showing various shapes and types of gold nanoparticles (AuNPs).

AuNPs display remarkable diversity in their size and shape (Figure 1), ranging from spherical, rod-shaped, triangular, and beyond. This variability stems from the various synthetic methods tested, such as seed-mediated growth, chemical reduction, or templated synthesis (Jana et al., 2001). Importantly, the size and shape of AuNPs profoundly influence their optical, electronic, and catalytic properties. For instance, smaller nanoparticles tend to exhibit a more intense and tunable surface plasmon resonance (SPR) absorption peak in the visible to near-infrared (NIR) range, making them particularly valuable for biomedical imaging and photothermal therapy applications (Dreaden et al., 2012). AuNP size and shape are crucial for their antimicrobial activity (Shamaila et al., 2016; Osonga et al., 2020). Smaller AuNPs are generally more effective due to a larger surface area to volume ratio, leading to stronger membrane interactions and easier cell entry, ultimately disrupting bacterial membranes and causing cell death (Shamaila et al., 2016; Osonga et al., 2020). The shape also matters; different shapes (e.g., spherical, rod, star) interact differently with bacteria, with star-shaped AuNPs potentially piercing cell walls more effectively (Shamaila et al., 2016; Osonga et al., 2020). Shape also influences the mechanism of action, sometimes promoting ROS generation or disrupting bacterial processes (Osonga et al., 2020). Therefore, controlling AuNP size and shape is essential for optimizing their antimicrobial properties in nanomedicine.

The optical properties of AuNPs are primarily governed by SPR, a phenomenon arising from the interaction of incident light with the free electrons at the metal surface. This collective electron oscillation results in a characteristic absorption and scattering peak, typically in the visible and near-infrared regions (Giljohann and Mirkin, 2009). The SPR properties, and consequently the color of colloidal AuNP solutions, can be precisely tuned by controlling factors such as size, shape, composition, and the surrounding medium, enabling tailored optical responses and facilitating qualitative analysis (Murphy et al., 2005). Optical properties are employed in cancer research, with both photothermal and photodynamic therapies demonstrating promising outcomes in cancer treatment using AuNPs (Eker et al., 2024).

Surface functionalization plays a pivotal role in modulating the physicochemical properties of AuNPs to suit specific applications. Functional ligands, such as thiolates, amines, or polymers, can be attached to the nanoparticle surface to enhance stability, solubility, and biocompatibility (Khan et al., 2019). Furthermore, surface modification enables the introduction of functional groups or biomolecules for targeted binding, facilitating applications in drug delivery, biosensing, and biocatalysis. The surface chemistry of AuNPs is crucial in determining their interaction with bacteria, with factors such as surface charge and potential being important (Saed et al., 2024). The surface chemistry of AuNPs can be tailored through specific synthesis methods and molecular binding, involving polymers, ligands, or biomolecules. These modifications significantly influence the nanoparticles’ electrostatic interactions with bacteria (Saed et al., 2024). Additionally, the surface chemistry is essential for the effective functionalization of AuNPs with antibiotics, potentially enhancing their antibacterial properties (Wang et al., 2014).

While gold is renowned for its chemical inertness, AuNPs can undergo surface oxidation under certain conditions, leading to changes in their properties and reactivity. Factors such as pH, temperature, and the presence of oxidizing agents can influence the extent of nanoparticle oxidation (Dreaden et al., 2012). Understanding the mechanisms and kinetics of surface oxidation is crucial to mitigating undesired alterations in AuNPs’ properties, thereby ensuring their stability and performance in various applications. Previous studies have shown that storing purified AuNP suspensions in the dark at 4°C can extend their stability for up to 20 days (Balasubramanian et al., 2010). Additionally, AuNPs used in earlier research maintain their atomic form without undergoing oxidation, even after being aerosolized in air or exposed to temperatures as high as 500°C (Balasubramanian et al., 2010). Due to their stability, biocompatibility, and ease of manipulation, AuNPs are considered a promising material for antibacterial applications.

AuNPs exhibit remarkable catalytic activity due to their high surface area-to-volume ratio, as well as their unique electronic and geometric structures. This catalytic ability spans a wide range of reactions, including oxidation, reduction, and hydrogenation (Khan et al., 2019). Notably, AuNPs are efficient catalysts in environmentally significant processes such as pollutant degradation, hydrogen production, and carbon dioxide reduction (Ghorbani-Vaghei et al., 2021). The tunability of AuNPs’ catalytic activity, achieved through adjustments in size, shape, and surface modification, holds promise for enhancing reaction selectivity and efficiency in various catalysis-driven industries. The catalytic activity of AuNPs, particularly their ability to generate ROS through redox reactions, plays a crucial role in their antimicrobial properties by inducing oxidative stress and disrupting bacterial cell membranes (Saed et al., 2024).

AuNPs are widely recognized for their inherent biocompatibility, which is attributed to their inert nature and low cytotoxicity. These properties make them highly suitable for various biomedical applications, including drug delivery, imaging, and therapy (Wang et al., 2014). Functionalization with biomolecules such as antibodies, peptides, or nucleic acids enables targeted delivery and enhanced imaging in biological systems, improving the precision of diagnostic and therapeutic approaches (Tang et al., 2020). Additionally, AuNPs can be readily internalized by cells and interact with biomolecules, further highlighting their potential for advancing healthcare technologies. The synthesis method plays a crucial role in tailoring nanoparticle properties for specific applications, as precise control over size, shape, and morphology directly influences their optical, electronic, catalytic, and biocompatibility characteristics (Niżnik et al., 2024). Advances in synthesis techniques have significantly expanded the versatility of AuNPs, fostering new applications across nanomedicine, diagnostics, and catalysis.

### 2.2 Synthesis methods of AuNPs

The chemical reduction method is one of the most widely used techniques for synthesizing AuNPs due to its simplicity, cost-effectiveness, and ability to control nanoparticle size and shape (Haiss et al., 2007; Sardar et al., 2009). In this approach, a gold precursor, typically chloroauric acid (HAuCl_*4*_), is reduced by a chemical reducing agent in the presence of a stabilizing agent to prevent aggregation. Common reducing agents include sodium borohydride (NaBH_*4*_), sodium citrate, and ascorbic acid, each influencing the reduction kinetics and final nanoparticle properties. The choice of reducing agent, along with reaction parameters such as temperature, pH, and reactant concentrations, plays a crucial role in governing nanoparticle nucleation and growth, thereby controlling the size and morphology of the resultant AuNPs (Darwish et al., 2022). By fine-tuning these parameters, researchers can synthesize AuNPs with diverse morphologies, including spherical, rod-shaped, triangular, and more complex structures, which are essential for various biomedical and catalytic applications (Niżnik et al., 2024).

Another method for synthesis is the seed-mediated growth method. This method is a widely utilized technique for achieving precise control over the size and shape of AuNPs by using pre-formed nanoparticles as seeds for further growth (Grzelczak et al., 2020). Initially, small, typically spherical AuNPs are synthesized as seeds via chemical reduction methods. These seeds are then introduced into a growth solution containing a gold precursor, a reducing agent, and specific additives such as surfactants or capping agents, which help regulate particle stability and morphology (Xia et al., 2009). By carefully adjusting reaction parameters—including the concentration of reactants, temperature, and reaction time—the final size and shape of the nanoparticles can be finely tuned. This method enables the synthesis of AuNPs with well-defined morphologies, including spheres, rods, cubes, and more intricate structures (Jana et al., 2001; Xia et al., 2009).

Additionally, template-assisted synthesis is a versatile technique that employs structured templates or molds to precisely control the size and shape of AuNPs (Gangwar et al., 2024). Templates can be inorganic, such as silica or anodic aluminum oxide (AAO), or organic, including polymers and biomolecules, which guide the nanoparticle growth process. The gold precursor is deposited onto the template surface, followed by chemical or electrochemical reduction, resulting in nanoparticles that conform to the template’s morphology (Gangwar et al., 2024). After synthesis, the template is typically removed via chemical dissolution or thermal treatment, leaving behind nanoparticles with well-defined sizes and shapes (Sperling and Parak, 2010; Chanana et al., 2016).

Another method is electrochemical methods, which provide a highly versatile and controllable approach for synthesizing AuNPs with well-defined size and morphology. In these methods, gold ions (Au^3+^ or AuCl_4_^−^) are reduced electrochemically at the electrode surface in an aqueous solution containing appropriate electrolytes and stabilizers (De Rooij, 2003). By precisely adjusting key parameters such as the applied potential, current density, electrolyte composition, and electrode geometry, researchers can fine-tune the nucleation and growth kinetics of AuNPs, thus controlling their size, shape, and distribution (Ramachandran et al., 2024). Additionally, the use of surfactants or capping agents helps regulate particle stability and prevent aggregation, further enhancing the reproducibility and uniformity of AuNP synthesis (De Rooij, 2003).

## 3 Mechanisms of antimicrobial action

**FIGURE 2:**
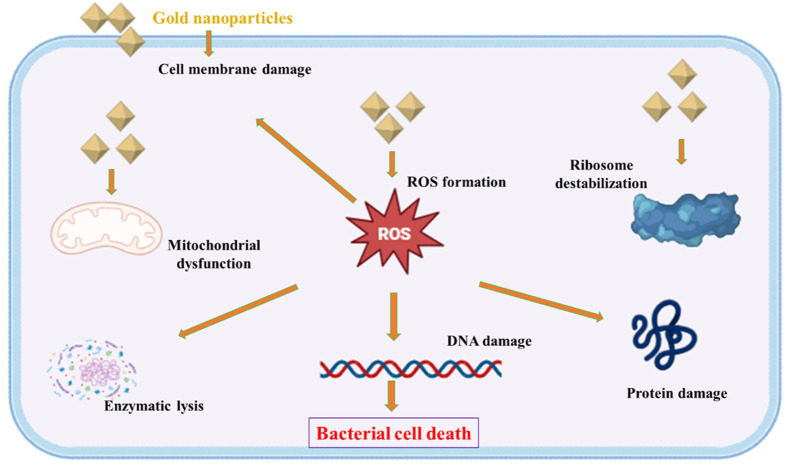
Schematic illustration of the antibacterial action of AuNPs. These mechanisms include direct interaction with pathogens, generation of oxidative stress, interference with essential cellular processes, and structural damage to the cell wall, DNA, and proteins.

AuNPs have accumulated attention for their remarkable ability to combat a wide range of pathogens, bacteria, fungi, and viruses. These mechanisms involve direct physical interactions with pathogens, oxidative stress induction, disruption of vital cellular functions, and damage to the cell wall, DNA, and proteins (Figure 2) (Akintelu, et al., 2021). It is worth to mention that, the antimicrobial activity of AuNPs is strongly influenced by their size, shape, and surface properties, which determine their interactions with microbial cells and their overall antimicrobial efficacy (Timoszyk and Grochowalska, 2022). Smaller-sized AuNPs, with their larger surface area, are more likely to exhibit enhanced antimicrobial effects due to increased cellular uptake and better interaction with microbial membranes (Timoszyk and Grochowalska, 2022). In addition, the shape of AuNPs, such as nanospheres or nanorods, plays a key role in influencing their ability to interact with microbial cells, with different shapes leading to varying mechanisms of action (Saed et al., 2024). Furthermore, surface modifications, including functionalization with antimicrobial agents, peptides, or targeting ligands, can enhance both the specificity and potency of AuNPs against a wide range of pathogens, thus making them more effective for targeted antimicrobial therapy (Saed et al., 2024). The antimicrobial effects of AuNPs primarily occur through three key mechanisms: direct interaction with microbial cells, generation of oxidative stress, and disruption of essential cellular processes.

### 3.1 Physical interaction with microbial cells

AuNPs exhibit antimicrobial activity primarily through direct physical interactions with microbial cells, leading to membrane disruption, oxidative stress, and intracellular disturbances (Akintelu, et al., 2021; Timoszyk and Grochowalska, 2022). Their high surface area-to-volume ratio enhances adsorption onto microbial cell membranes, destabilizing lipid bilayers and increasing membrane permeability (Timoszyk and Grochowalska, 2022). This disruption compromises membrane integrity, resulting in leakage of cytoplasmic contents and loss of cellular homeostasis, ultimately leading to bacterial cell death (Timoszyk and Grochowalska, 2022).

### 3.2 Induction of oxidative stress

AuNPs contribute to oxidative stress within microbial cells by inducing the generation of reactive oxygen species (ROS), including superoxide radicals, hydroxyl radicals, and singlet oxygen (Fu et al., 2014). These ROS cause oxidative damage to essential biomolecules such as lipids, proteins, and nucleic acids, leading to cellular dysfunction and loss of viability (Juan et al., 2021). In bacteria, ROS disrupt membrane integrity and interfere with enzymatic pathways, while in eukaryotic microbes, they may impair mitochondrial function (Juan et al., 2021). The overwhelming oxidative stress ultimately compromises microbial survival and enhances the antimicrobial efficacy of AuNPs (Fu et al., 2014).

### 3.3 Inhibition of cellular processes

AuNPs disrupt essential microbial cellular processes, including DNA replication, transcription, and protein synthesis, thereby inhibiting growth and proliferation (Akintelu et al., 2021; Mikhailova, 2021). By binding to nucleic acids and proteins, AuNPs can induce conformational changes, disrupting enzymatic activities and ribosomal functions, which hampers microbial metabolism and impedes cellular replication (Akintelu et al., 2021). These interactions interfere with critical cellular machinery such as DNA polymerases and ribosomal subunits, preventing efficient protein synthesis and DNA replication. This disruption of vital cellular processes enhances the antimicrobial efficacy of AuNPs against a broad range of pathogens, including bacteria, fungi, and viruses (Wang et al., 2017; Mikhailova, 2021).

## 4 Antimicrobial spectrum of gold nanoparticles

AuNPs have demonstrated significant antimicrobial activity against various MDR pathogens, including bacteria, viruses, and fungi, positioning them as potential alternatives to traditional antibiotic therapies. Their unique physicochemical properties allow them to combat bacterial resistance mechanisms, such as efflux pumps, biofilm formation, and quorum sensing inhibition, making them effective against antibiotic-resistant strains. Studies have shown their efficacy against a wide range of pathogens, as demonstrated in Table 1. For instance, they have shown efficacy against methicillin-resistant *Staphylococcus aureus* (MRSA), where AuNP-conjugated berberine achieved a lower MIC (109.5 μg/mL) compared to free berberine (165 μg/mL), along with higher biofilm eradication (22.33% vs. 13.9%) and increased ROS generation leading to membrane disruption (Sadeghi et al., 2024). They are also effective against vancomycin-resistant *Enterococcus* (VRE), where AuNPs immobilized with vancomycin, when combined with near-infrared light, reduced the required antibiotic concentration 16-fold (Wang et al., 2018), and *Escherichia coli*, where AuNPs triggered apoptosis-like cell death via glutathione depletion and DNA damage without disrupting cytoplasmic membrane integrity (Lee and Lee, 2018). Similarly, *Pseudomonas aeruginosa* exhibited reduced biofilm formation at sub-MICs (50–150 μg/mL) (Ali S. et al., 2020; Ali S. G. et al., 2020), and in *Acinetobacter baumannii*, AuNPs functionalized with DNA aptamers and AMPs effectively cleared infections in mice (Park et al., 2022). Against *Mycobacterium tuberculosis*, biosynthesized AuNPs showed strong efficacy with MIC_99_ of 6.42 μg/mL, and several tested nanoconjugates exhibited exceptional anti-TB activity (Priya et al., 2023). Furthermore, *Streptococcus pneumoniae* was inhibited by Arthrospira platensis-mediated AuNPs, which outperformed tigecycline with a MIC of 12 μg/mL (Azmy et al., 2024), and *Klebsiella pneumoniae* biofilms were disrupted by chlorhexidine-coated AuNPs, which also eliminated preformed biofilms (Ahmed et al., 2016). AuNPs exert their antibacterial effects through multiple mechanisms, including bacterial membrane disruption, inhibition of key enzymatic functions, interference with DNA replication, and induction of oxidative stress, leading to microbial cell death (Lee and Lee, 2018; Ali S. et al., 2020; Ali S. G. et al., 2020; Saed et al., 2024; Sadeghi et al., 2024).

**TABLE 1:** Summary of key studies on the antimicrobial activity of gold nanoparticles.

Pathogen tested	Antimicrobial activity	Potential mechanism of action	References
MRSA	The AuNP-conjugated berberine showed a lower MIC against MRSA (109.5 μg/mL) compared to free berberine (165 μg/mL). At their respective MIC levels, both free and conjugated berberine exhibited anti-biofilm activity, achieving biofilm eradication rates of 13.9% and 22.33%, respectively	A significant increase in reactive oxygen species (ROS) production (93%) was observed with conjugated BER at a concentration of 27.37 μg/mL, leading to cell membrane disruption and decreased bacterial viability	Sadeghi et al. (2024)
Vancomycin-resistant *Enterococcus*	Polygonal-shaped gold nanoparticles immobilized with vancomycin demonstrate both antibacterial activity and photothermal efficacy against VRE. The Au@Van NPs, when combined with near-infrared light irradiation for 5 min, require 16 times less vancomycin to inhibit vancomycin-resistant Enterococci compared to free vancomycin	Not determined	Wang et al. (2018)
*Escherichia coli*	AuNPs exhibit antibacterial activity without compromising the integrity of the cytoplasmic membrane	AuNPs induce apoptosis-like cell death in *Escherichia coli* by disrupting redox balance through glutathione depletion. They also penetrate the cell membrane, causing damage to DNA and other nucleic acids	Lee and Lee (2018)
*Pseudomonas aeruginosa*	The sub-minimum inhibitory concentrations (50, 100, and 150 μg/mL) of AuNPs significantly impacted the biofilm formation of *P. aeruginosa*	Not determined	Ali et al. (2020a), Ali et al. (2020b)
*Acinetobacter baumannii*	Lys AB2 P3-His, a hexahistidine-tagged antimicrobial peptide (AMP) loaded onto DNA aptamer-functionalized gold nanoparticles (AuNP-Apt), effectively inhibits A. baumannii infection in mice	Not determined	Park et al. (2022)
*Mycobacterium tuberculosis*	The biosynthesized gold nanoparticles and nanoconjugates exhibited a minimum inhibitory concentration that achieved 99% inhibition (MIC_99_) of 6.42 μg/mL. Among the 15 nanoparticles tested, seven (NP1, NP2, NP6, NP7, NP10, NP12, and NP15) demonstrated exceptional anti-TB activity, while the remaining nanoparticles displayed varying levels of inhibition	Not determined	Priya et al. (2023)
*Streptococcus pneumoniae*	The synthesized *Arthrospira platensis*-mediated AuNPs demonstrated strong antibacterial activity against *S. pneumoniae*, with a MIC of 12 μg/mL, outperforming the control antibiotic, tigecycline	Not determined	Azmy et al. (2024)
*Klebsiella pneumoniae*	Chlorhexidine-coated gold nanoparticles not only prevent biofilm formation by *K. pneumoniae* ATCC and clinical isolates but also eliminate preformed biofilms	Not determined	Ahmed et al. (2016)
*Candida* spp.	The synthesized caspofungin-coated AuNPs significantly lowered the minimum inhibitory concentration against *C. albicans* (P = 0.0005) and non-albicans *Candida* (NAC) species (P < 0.0001)	SEM analysis results confirmed the impact of AuNPs on the cell wall structure of *C. glabrata*, leading to the formation of pores	Salehi et al., 2021
*Aspergillus fumigatus*	The green-synthesized gold nanoparticles, using the ethanolic leaf extract of *Leptadenia hastata*, exhibited antifungal activity against *Aspergillus fumigatus*, with a MIC of 64 μg/mL, and reduced the radial growth of *A. fumigatus* by 30% compared to the control	Deformation and collapse of fungal hyphae, along with degradation of cell walls	Abdallah and Ali (2022)
*Cryptococcal neoformans*	In mice infected with *C. neoformans*, five daily treatments with amphotericin B complexed with gold nanoparticles (containing 0.25 mg/kg AmB) significantly reduced the fungal burden in brain tissue compared to untreated mice or those treated with 0.25 mg/kg of AmB alone	Not determined	Chintalacharuvu et al., 2021
Influenza virus	AuNPs inhibited virus replication in a dose-dependent manner, with the lowest concentration (0.06 μg/mL) leading to a 2–3.5 log10 TCID50/mL reduction in virus output	Not determined	Babaei et al. (2021)
Herpes simplex virus (HSV)	AuNPs can reduce the cytopathic effect (CPE) of HSV-1 in Vero cells in a dose- and time-dependent manner when applied in pretreatment mode. The observed antiviral activity occurred within the nontoxic concentration range of AuNPs	The observed effect could potentially be attributed to the localized effects of nanoparticles on the virus envelope	Paradowska et al. (2021)
RNA viruses	AuNPs had a significant inhibitory effect on both lentivirus and HCoV-OC43	AuNPs, especially those with positive surface charges, likely exert their antiviral effects by modifying lysosomal function and organelle dynamics, thereby interfering with viral entry, replication, or release	Li et al. (2023a), Li et al. (2023b)

Additionally, AuNPs have shown antifungal properties against drug-resistant fungi, such as *Candida* species, where caspofungin-coated AuNPs significantly lowered the MIC against both *C. albicans* and non-*albicans* species, as confirmed by SEM analysis showing pore formation in fungal cell walls (Salehi et al., 2021); *Aspergillus fumigatus*, where green-synthesized AuNPs reduced radial growth by 30% and showed a MIC of 64 μg/mL due to hyphal collapse and wall degradation (Abdallah and Ali, 2022); and *Cryptococcal neoformans*, where gold nanoparticle-AmB complexes (0.25 mg/kg) significantly reduced fungal burden in mouse brain tissue compared to free drug or untreated controls (Chintalacharuvu et al., 2021).

Beyond their antibacterial and antifungal properties, AuNPs have exhibited potent antiviral activity against various viruses. Against influenza virus, even a low dose of 0.06 μg/mL led to a 2–3.5 log_10_ TCID_50_/mL reduction in viral output (Babaei et al., 2021); with herpes simplex virus (HSV), AuNPs reduced cytopathic effects in Vero cells in a dose- and time-dependent manner during pretreatment (Paradowska et al., 2021); and for RNA viruses such as lentivirus and HCoV-OC43, positively charged AuNPs disrupted viral replication by interfering with lysosomal function and organelle dynamics (Li F. et al., 2023; Li Y. et al., 2023). Their antiviral effects primarily stem from their ability to directly interact with viral envelope proteins, alter lysosomal function, and affect organelle dynamics, thereby hindering viral entry, replication, and release (Paradowska et al., 2021; Li F. et al., 2023; Li Y. et al., 2023).

## 5 Synergistic effects of AuNPs with antibiotics

AuNPs have been utilized to enhance the antibacterial efficacy of various antibiotics by altering their properties and interactions with bacterial cells. Several studies have demonstrated that conjugating antibiotics with AuNPs improves their antimicrobial potency, often by reducing the minimum inhibitory concentration (MIC) compared to the free drug (Table 2).

**TABLE 2:** Enhanced antibacterial activity of antibiotic-gold nanoparticle conjugates.

Antibiotic	Class	Gold nanoparticle (AuNP) characteristics	Synthesis and optical properties	Tested bacteria	Minimum inhibitory concentration (MIC) of AuNP conjugate	Key findings	References
Amoxicillin	β-lactams	Irregular (triangular, hexagonal, spherical)	18 min, 50°C	*P. aeruginosa, S. aureus*	1.5 μg/mL	60%–70% biofilm viability reduction	Rocca et al. (2020)
Amoxicillin		Hexagonal/spherical, 15.99-24.71 nm	1 h, 25°C; 534 nm UV peak	MRSA*, E. coli*	3.6–8 μg/mL	12-31x MIC reduction vs. amoxicillin alone	Halawani et al. (2023)
Ampicillin		1.43 ± 0.5 nm	24 h, room temp	MRSA, *S. aureus*	0.58 μg/mL (*S. aureus*); 4 μg/mL (MRSA)	18% MIC reduction *(S. aureus*); 10-20x reduction (MRSA)	Fan et al. (2019)
6-Amino-penicillanic acid		∼3 nm	∼1 h, ice water bath	*E. coli, K. pneumoniae, P. aeruginosa,* MDR *E. coli*, MDR *K. pneumoniae*	2.5 μg/mL (*E. coli*); 5 μg/mL (*K. pneumoniae*); 1 μg/mL (others)	Significant MIC reduction, especially in MDR strains	Yang et al. (2017)
Cefixime		Spherical, 25–50 nm	2.5 h; 532 nm UV peak	*S. aureus*	45 ± 0.12 μg/mL (3.24 μg cefixime equiv.)	8x increase in cefixime efficacy	Ali et al. (2020a), Ali et al. (2020b)
Cefotaxime		Spherical, monodispersed; 6.87 ± 2.43 nm (AuNP), 17.55 ± 2.95 nm (conjugate)	48 h, 40°C; 542 nm UV peak	*E. coli, K. pneumoniae*	1.009 μg/mL (*E. coli*); 2.018 μg/mL (*K. pneumoniae*)	AuNPs gained antibacterial activity via conjugation	Shaikh et al. (2017)
Cefoxitin		Spherical, polydispersed, 2–12 nm	48 h, 40°C; 518 nm UV peak	*E. coli, K. pneumoniae*	1.5 μg/mL (*E. coli*); 2.5 μg/mL (*K. pneumoniae*)	AuNPs enhanced drug delivery, restored efficacy	Alafnan et al. (2022)
Imipenem/Meropenem		35-200 nm	20 min; 530 nm UV peak	*K. pneumoniae, P. mirabilis, A. baumannii*	2.5 μg/mL (*K. pneumoniae*); ∼1.25 μg/mL (*P. mirabilis*, A. baumannii)	4x MIC reduction (imipenem); 3x reduction (meropenem)	Shaker and Shaaban (2017)
Amikacin	Aminoglycoside	Spherical; 3.3 nm (citrate-AuNP), 11.5 nm (PVP-AuNP), 6.25 nm (Tween 20-AuNP)	2 h, room temp; 533/537/535 nm UV peaks	*E. coli, S. aureus*	-	Enhanced activity vs. amikacin alone (all surfactants)	Kaur and Kumar (2022)
Vancomycin	Glycopeptide	Spherical, monodispersed, 24 nm	48 h, 40°C; 524 nm UV peak	*E. coli, K. oxytoca, P. aeruginosa, S. aureus*	93.44 μg/mL (*E. coli*); 70.84 μg/mL (K*. oxytoca*); 60.65 μg/mL (*P. aeruginosa*); 30.63 μg/mL (*S. aureus*)	1.4-1.8x activity increase	Hagbani et al. (2022)
Doxycycline	Tetracycline	Spherical, 13 ± 1.2 nm	15 min; 540 nm UV peak	*S. aureus, E. coli, K. pneumoniae, A. baumannii, P. aeruginosa*	2 μg/mL	∼16x MIC reduction vs. doxycycline alone	Fuller et al. (2020)
Colistin	Polymyxins	-	-	*E. coli*	0.23 ± 0.03 μg/mL	6.8x MIC reduction	Haddada et al. (2018)

This synergistic effect was studied with different classes of antibiotics. For instance, in the case of β-lactams, amoxicillin was synthesized into AuNPs of irregular (triangular, hexagonal, and spherical) and hexagonal/spherical shapes. The conjugates exhibited significant biofilm reduction in *S. aureus* (60%) and *Pseudomonas aeruginosa* (70%) (Rocca et al., 2020). Moreover, in another study, amoxicillin-coated AuNPs (15.99–24.71 nm) demonstrated MIC values 12–31 times lower than free amoxicillin against methicillin-resistant *S. aureus* (MRSA) and *E. coli* (Halawani et al., 2023). Ampicillin-loaded AuNPs (1.43 ± 0.5 nm) showed enhanced antibacterial activity, reducing the MIC by 18% against *S. aureus* and by 10–20 times against MRSA compared to ampicillin alone (Fan et al., 2019). Additionally, 6-amino-penicillanic acid-coated AuNPs (∼3 nm) reduced the MIC from over 250 μg/mL to 5 μg/mL against MDR *E. coli* and *K. pneumoniae* (Yang et al., 2017). Cefixime, another cephalosporin β-lactam antibiotic, was conjugated with spherical AuNPs (25–50 nm) and demonstrated an eightfold increase in efficiency against *Staphylococcus aureus* with an MIC of 45 ± 0.12 μg/mL (3.24 μg cefixime) (Ali S. et al., 2020; Ali S. G. et al., 2020). Similarly, cefotaxime-coated AuNPs (6.87–17.55 nm) exhibited potent antibacterial activity against *Escherichia coli* and *Klebsiella pneumoniae*, with MIC values of 1.009 μg/mL and 2.018 μg/mL, respectively, whereas pure AuNPs lacked antibacterial effects (Shaikh et al., 2017). Cefoxitin, another cephalosporin, showed improved delivery efficiency when conjugated with AuNPs (2–12 nm), transforming it from an ineffective antibiotic into a responsive antimicrobial agent against *E. coli* and *K. pneumoniae* (Alafnan et al., 2022). Carbapenem antibiotics, including imipenem and meropenem which are considered the last resort for treatment of highly resistant bacteria (Khalifa et al., 2020a; Khalifa et al., 2020b; Khalifa et al., 2020c), were synthesized with AuNPs (35–200 nm) and displayed reduced MIC values against *K. pneumoniae*, *P. mirabilis*, and *A. baumannii*. The MIC of imipenem decreased by fourfold, while meropenem’s MIC was reduced threefold (Shaker and Shaaban, 2017).

Aminoglycoside and glycopeptide another group of antibiotics also benefited from AuNP conjugation. Amikacin-coated AuNPs (3.3–11.5 nm) exhibited improved antibacterial activity against *E. coli* and *S. aureus* compared to free amikacin (Kaur and Kumar, 2022). Vancomycin-conjugated AuNPs (24 nm) enhanced antimicrobial effects by 1.4–1.8 times against *E. coli*, *K. oxytoca*, *S. aureus*, and *P. aeruginosa* (Hagbani et al., 2022). Tetracycline antibiotics, such as doxycycline, showed remarkable efficacy improvement when conjugated with AuNPs (13 ± 1.2 nm), reducing the MIC by nearly 16 times against *S. aureus*, *E. coli*, *K. pneumoniae*, *A. baumannii*, and *P. aeruginosa* (Fuller et al., 2020). Similarly, colistin-loaded AuNPs significantly reduced the MIC by 6.8-fold against *E. coli* (Haddada et al., 2018). These findings collectively highlight the potential of AuNP-antibiotic conjugates in enhancing antibacterial efficacy, reducing required dosages, and overcoming resistance mechanisms in various bacterial strains.

Beyond bacterial infections, synergistic interactions have also been documented against viral and fungal pathogens. Recent findings by Dasilva et al. (2024) highlight the enhanced antifungal efficacy of Au NPs when combined with amphotericin B, particularly against persister cells within *Candida tropicalis* biofilms (Dasilva et al., 2024). This combination significantly improved antifungal activity, overcoming the inherent resistance of biofilms and effectively reducing fungal viability. These findings are particularly significant given the rising global threat of antifungal resistance (Khalifa et al., 2022a; Khalifa et al., 2022b; Khalifa et al., 2023). This approach offers a promising new strategy to combat fungal resistance effectively (Khalifa et al., 2024a). Furthermore, Malik et al. developed a topical gel as a vaginal microbicide by incorporating AuNPs with the antiretroviral drug efavirenz (Malik et al., 2018). This formulation effectively inhibited P24 and exhibited a favorable safety profile regarding toxicity. Furthermore, drug susceptibility assays revealed a synergistic effect of AuNPs, as the combination of efavirenz and AuNPs led to a greater reduction in P24 production compared to efavirenz alone (Malik et al., 2018).

## 6 Targeted delivery systems

The development of targeted drug delivery systems has revolutionized antimicrobial therapy by enhancing drug efficacy and minimizing off-target effects. AuNPs have emerged as promising carriers for targeted drug delivery due to their biocompatibility, tunable surface chemistry, and ability to improve drug pharmacokinetics (Wang et al., 2014). Functionalization of AuNPs with antimicrobial agents, targeting ligands, and surface coatings has facilitated precision drug delivery to MDR pathogens, enhancing therapeutic outcomes while reducing toxicity (Li et al., 2014). Several strategies have been employed to optimize AuNP-based targeted delivery systems against MDR pathogens. These strategies include antibody-mediated targeting, aptamer-functionalization, and ligand-conjugation (Table 3). Each approach aims to enhance the specificity and efficacy of antimicrobial agents against resistant bacterial and fungal strains.

**TABLE 3:** Summary of targeted drug delivery strategies using AuNPs.

Strategy	Functionalization method	Target pathogen	Key advantage	References
Antibody-Mediated Targeting	Pathogen-specific antibodies	*S. aureus*	Enhanced pathogen recognition	Kirui et al. (2019)
Aptamer-Functionalized AuNPs	DNA/RNA aptamers	*E. coli* and *S. aureus*	High specificity and affinity for detection of the pathogenes	Zhang et al. (2018)
Ligand-Conjugated AuNPs	Peptides	*Pseudomonas aeruginosa*	Enhanced the peptide’s activity without showing toxicity to human cells	Casciaro et al. (2017)

### 6.1 Antibody-mediated targeting

**FIGURE 3:**
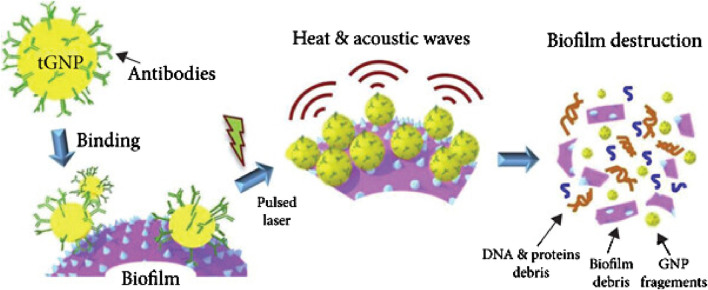
The mechanism by which AuNPs disperse biofilms via photothermal therapy involves several key steps. Initially, GNPs functionalized with pathogen-specific antibodies (e.g., anti-*Staphylococcus aureus* antibodies) selectively bind to biofilms formed by the target microorganisms. Upon exposure to pulsed laser irradiation, the GNPs absorb the energy and generate localized heat along with acoustic waves. The resulting thermal and mechanical effects disrupt the biofilm structure, leading to its dispersion. Reproduced with permission from Kirui et al., 2019, under license CC BY-NC-ND 4.0, copyright 2019 Elsevier B.V.

The conjugation of AuNPs with pathogen-specific antibodies enables selective binding to bacterial or fungal cells. Additionally, the combination of antibody targeting with gold nanoparticle photothermal therapy allows for an immunologically mediated destruction of bacteria. For example, *S. aureus* and its biofilms can be effectively eliminated through this dual mechanism (Figure 3) (Kirui et al., 2019).

### 6.2 Aptamer-functionalized AuNPs

Aptamers, which are short single-stranded DNA or RNA molecules with high specificity for bacterial surface markers, have been utilized to functionalize AuNPs for pathogen detection. This approach enables the precise identification of bacterial contaminants. For instance, Zhang et al. developed a dual-recognition system that combines vancomycin and aptamers to simultaneously detect *Escherichia coli* and *Staphylococcus aureus* (Zhang et al., 2018). In this system, vancomycin is incorporated into Fe_3_O_4_@Au nanoparticles, serving as a broad-spectrum bacterial capture agent that effectively concentrates the target microorganisms. Aptamer-functionalized AuNPs, along with two distinct types of surface-enhanced Raman scattering (SERS) tags, are then employed for highly sensitive and specific quantitative analysis. This innovative platform achieves detection limits as low as 20 cells/mL for *S. aureus* and 50 cells/mL for *E. coli*, demonstrating its potential for rapid and accurate bacterial identification.

### 6.3 Ligand-conjugated AuNPs

**FIGURE 4:**
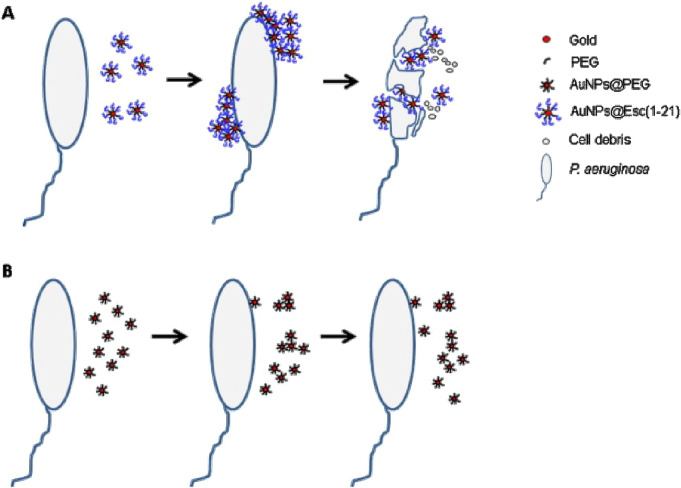
Illustration of the Mode of Action of AuNPs@Esc(1-21) and AuNPs@PEG on *P. aeruginosa.* The figure depicts the interaction of AuNPs@Esc(1-21) **(A)** and AuNPs@PEG **(B)** with *Pseudomonas aeruginosa*. Upon contact with bacteria, AuNPs@Esc(1-21) rapidly accumulate at various sites on the bacterial surface without penetrating the cells, leading to structural disruption. In contrast, while AuNPs@PEG tend to form clusters in solution, they remain inactive against *Pseudomonas* and do not attach to the bacterial surface. Reproduced with permission (license number 5984611123801) Casciaro et al. (2017), copyright 2017 Elsevier B.V.

Surface modification of AuNPs with ligands such as folic acid, transferrin, or peptides facilitates selective uptake by microbial cells. For instance, studies have demonstrated that functionalizing AuNPs with antimicrobial peptides can facilitate targeted delivery to bacterial cells, enhancing antimicrobial efficacy. For instance, Casciaro et al. demonstrated that the covalent attachment of Esc(1-21) to soluble AuNPs, forming AuNPs@Esc(1-21) through a poly(ethylene glycol) linker, enhanced the peptide’s activity by approximately 15 times against both motile and sessile forms of *Pseudomonas aeruginosa*, without showing toxicity to human keratinocytes (Casciaro et al., 2017). Additionally, AuNPs@Esc(1-21) exhibited significantly improved resistance to proteolytic degradation and was able to disrupt the bacterial membrane at very low concentrations (5 nM) (Figure 4) (Casciaro et al., 2017).

## 7 Resistance and biocompatibility

AuNPs have attracted considerable interest across multiple disciplines, including medicine, environmental science, and nanotechnology, due to their distinct physicochemical properties. However, concerns have been raised regarding the potential emergence of microbial resistance to AuNPs, which could undermine their effectiveness in various applications. Over the past five years, extensive research has investigated the mechanisms of microbial resistance to nanoparticles such as AgNPs and zinc oxide nanoparticles (ZnONPs) (Kamat and Kumari, 2023). Studies have elucidated key factors contributing to resistance development, including nanoparticle size, surface modifications, and species-specific microbial responses (Kamat and Kumari, 2023). Despite these findings, no study to date has reported the development of antimicrobial resistance against AuNPs.

The absence of reported microbial resistance to AuNPs can be attributed to their distinct mechanisms of antimicrobial action, which differ significantly from conventional antibiotics and other metal nanoparticles. Unlike AgNPs and ZnONPs, which primarily exert toxicity through ion release, oxidative stress, and membrane disruption, AuNPs employ multiple antimicrobial pathways that make resistance development highly unlikely (Lee and Lee, 2018; Sadeghi et al., 2024). AuNPs can interfere with bacterial metabolic pathways, and disrupt essential cellular functions preventing bacteria from effectively neutralizing their effects (Wang et al., 2017; Lee and Lee, 2018; Sadeghi et al., 2024). Moreover, their ability to be functionalized with antimicrobial peptides, antibiotics, or targeting ligands enhances their selectivity and potency against MDR pathogens (Casciaro et al., 2017; Zhang et al., 2018; Kirui et al., 2019). This multi-target approach significantly reduces the likelihood of bacteria evolving resistance compared to other nanoparticles that rely on a single mode of action. However, ongoing research is essential to monitor the potential development of resistance and devise strategies to prevent its emergence.

Addressing the safety and biocompatibility of AuNPs is critical for their successful application across various fields. While AuNPs offer advantages such as unique optical properties, a high surface area-to-volume ratio, and ease of functionalization, assessing their potential adverse effects is essential for ensuring safe biomedical use. Several factors influence the biocompatibility of AuNPs, including particle size, shape, surface chemistry, and the route of administration. Studies indicate that smaller AuNPs exhibit higher cellular uptake, which may contribute to increased cytotoxicity compared to larger nanoparticles (Xia et al., 2019). Additionally, the shape of AuNPs—whether spherical, rod-shaped, or other geometries—affects their interactions with biological systems and can influence toxicity profiles (Niżnik et al., 2024). Surface chemistry, dictated by functional groups or coatings, plays a pivotal role in determining nanoparticle biocompatibility, immune responses, and overall safety (Li et al., 2020). Moreover, biological barriers, such as the blood-brain barrier and cellular membranes, significantly impact the biodistribution and potential toxicity of AuNPs. Understanding these interactions is crucial for predicting nanoparticle behavior *in vivo* and optimizing their design for clinical applications. The route of administration—whether intravenous, oral, dermal, or inhalation—also affects bioavailability, systemic circulation, and toxicity (Chenthamara et al., 2019). Comprehensive biocompatibility assessments are essential to ensure the safe use of AuNPs. These include *in vitro* and *in vivo* evaluations of cellular uptake, cytotoxicity, genotoxicity, immunotoxicity, and organ-specific toxicity (Kumar et al., 2017; Lama et al., 2020; Kus-Liśkiewicz et al., 2021). Additionally, long-term pharmacokinetic studies investigating the biodistribution, metabolism, and clearance of AuNPs are crucial for defining their safety profiles and mitigating potential risks. By integrating these evaluations, researchers can develop safer AuNP-based formulations with minimal adverse effects, thereby maximizing their therapeutic potential.

## 8 Safety concerns associated with AuNPs

Despite the promising potential of AuNPs in various biomedical applications, there are notable safety challenges that must be carefully addressed to ensure their responsible clinical deployment. The physicochemical characteristics of AuNPs—including their size, shape, surface chemistry, and charge—can significantly influence their biological interactions, leading to potential toxicity or unintended immune responses. These factors necessitate robust and standardized evaluation protocols to determine biocompatibility and mitigate health or environmental risks. To ensure safe clinical translation, the following key aspects must be prioritized.

### 8.1 Ensuring biocompatibility

Ensuring the biocompatibility and safety of AuNPs for clinical use remains a primary challenge. Despite their promising antimicrobial properties, rigorous safety assessments are necessary to mitigate potential adverse effects, such as cytotoxicity and immunogenicity, before their widespread clinical application (Kus-Liśkiewicz et al., 2021). These assessments are crucial for determining appropriate dosages and identifying any long-term risks associated with their use (Xuan et al., 2023).

### 8.2 Standardization of synthesis and characterization

Reproducibility in nanoparticle research is critically dependent on standardization. Inconsistent synthesis methods often lead to batch variability, affecting both therapeutic performance and safety outcomes. Therefore, establishing internationally accepted guidelines for nanoparticle fabrication, purification, and physicochemical characterization is essential to advance regulatory approval and clinical implementation (Amina and Guo, 2020; Tehrani et al., 2023).

### 8.3 Optimization of formulations and delivery methods

Tailoring AuNP formulations and delivery methods to specific clinical applications is a significant challenge. Optimizing parameters such as particle size, surface charge, and surface functionalization is critical for enhancing antimicrobial efficacy while minimizing off-target effects and improving bioavailability (Sibuyi et al., 2021). Effective formulations that enable targeted delivery can improve therapeutic outcomes and reduce potential toxicity to healthy tissues (Elumalai et al., 2024).

### 8.4 Regulatory hurdles and approval processes

Navigating regulatory hurdles and obtaining approval from agencies such as the FDA (Food and Drug Administration) for clinical use represents a substantial challenge. Demonstrating the safety, efficacy, and quality of AuNP-based antimicrobial products through preclinical studies and clinical trials is essential. This process requires considerable time, resources, and investment but is necessary to bring these advanced therapies to market (Allan et al., 2021; Ramos et al., 2022).

### 8.5 *In vivo* and clinical investigations

Extensive *in vivo* investigations are needed to assess the safety, pharmacokinetics, and efficacy of AuNPs in combating MDR infections. Preclinical studies using animal models can provide valuable insights into the biodistribution, tissue penetration, and therapeutic potential of AuNP-based antimicrobial agents (Xia et al., 2019; Lama et al., 2020). Furthermore, nanotoxicological studies must explore oxidative stress induction, mitochondrial disruption, and potential genotoxicity. Future research should aim at identifying biomarkers of nanoparticle exposure and developing predictive models of toxicity (Kumar et al., 2017; Chenthamara et al., 2019; Xia et al., 2019; Li et al., 2020; Lama et al., 2020; Kus-Liśkiewicz et al., 2021).

## 9 Future directions

AuNPs hold considerable promise in addressing multidrug-resistant (MDR) infections owing to their unique physicochemical and antimicrobial properties. However, translating these potentials into clinical solutions requires strategic advancements. Key future directions include fostering interdisciplinary collaboration, designing personalized therapeutic strategies, integrating AuNPs with conventional therapies, advancing synthesis and functionalization techniques, and expanding their biomedical utility beyond antimicrobial applications.

### 9.1 Multidisciplinary collaboration

Successful translation of AuNP-based antimicrobial technologies depends on robust collaboration among microbiologists, materials scientists, clinicians, regulatory authorities, and industry partners. Such cooperation will enhance the design of comprehensive evaluation protocols, streamline regulatory approval, and facilitate knowledge exchange to accelerate clinical translation (Malik et al., 2023; Fosse et al., 2023).

### 9.2 Tailored therapeutic strategies

The development of personalized and targeted therapeutic strategies using AuNPs holds significant promise for addressing antimicrobial resistance and improving treatment outcomes. Tailoring AuNP formulations and delivery methods to target specific pathogens or infection sites while minimizing systemic toxicity can enhance therapeutic efficacy and reduce the emergence of resistance (Makabenta et al., 2021; Kamalavarshini et al., 2023).

### 9.3 Integration with existing therapies

Integrating AuNP-based antimicrobial therapies with existing treatments, such as antibiotics or antifungals, provides synergistic benefits and helps address the limitations of conventional therapies (Ahmed et al., 2016; Chintalacharuvu et al., 2021; Salehi et al., 2021; Abdallah and Ali, 2022; Priya et al., 2023; Azmy et al., 2024). Additionally, combining AuNPs with other therapeutic approaches, such as photothermal therapy or immunotherapy, can enhance their antimicrobial effectiveness and expand their therapeutic potential (Songca, 2023; Kumar and Lim, 2023). Further research is needed to explore the dual benefits of AuNPs when combined with other treatments, aiming to minimize side effects and improve their overall therapeutic efficacy.

### 9.4 Translation into point-of-care applications

Developing AuNP-based antimicrobial products suitable for bedside testing, such as diagnostic assays or wound dressings, can facilitate rapid and decentralized management of infectious diseases (Pang et al., 2023). Portable and user-friendly AuNP-based technologies capable of detecting and treating microbial infections can transform clinical practice, particularly in resource-limited settings (Nath, et al., 2020; Gradisteanu Pircalabioru et al., 2024). Further advancements in AuNP-based antimicrobial therapies could facilitate their translation into point-of-care applications, enabling rapid and effective treatment of infections in clinical and field settings.

### 9.5 Synthesis methods

Recent progress in the field has emphasized the development of efficient and scalable synthesis techniques for gold nanoparticles (AuNPs), with precise control over their size and shape. Eco-friendly “green” synthesis methods—using plant extracts, microorganisms, or natural biomolecules—are increasingly favored for their sustainability and potential to yield AuNPs with superior antimicrobial activity (Niżnik et al., 2024). In parallel, advanced methods such as microwave-assisted and electrochemical synthesis offer fast, reproducible, and fine-tuned control over nanoparticle characteristics (Adeola et al., 2023). Moving forward, research should prioritize the creation of synthesis strategies that are not only environmentally sustainable and cost-effective but also capable of producing AuNPs with improved stability and enhanced antimicrobial efficacy.

### 9.6 Functionalization strategies

Surface functionalization of AuNPs plays a crucial role in improving their stability, biocompatibility, and targeting ability. Coating AuNPs with antimicrobial peptides, antibiotics, or polymers enhances their specificity towards MDR pathogens while minimizing off-target effects (Zhang et al., 2018; Kirui et al., 2019). Moreover, the conjugation of targeting ligands onto AuNPs enables selective interaction with pathogen-specific receptors, facilitating targeted drug delivery and enhanced therapeutic efficacy (Kamat and Kumari, 2023). Therefore, future research is essential to improve the functionalization of AuNPs.

### 9.7 Antimicrobial mechanisms

Elucidating the underlying mechanisms of AuNP-mediated antimicrobial activity is essential for optimizing their efficacy and minimizing potential resistance development. Studies suggest that AuNPs exert antimicrobial effects through multiple mechanisms including membrane damage, ROS generation, and disruption of cellular functions (Lee and Lee, 2018; Sadeghi et al., 2024). However, the exact mechanism of action must be thoroughly investigated to optimize their clinical application.

### 9.8 Biomedical applications

In addition to their antimicrobial properties, gold nanoparticles (AuNPs) hold significant promise across a broad range of biomedical applications, including diagnostics, imaging, and targeted drug delivery. When functionalized appropriately, AuNPs can be utilized as versatile platforms for developing rapid and highly sensitive diagnostic tools to identify multidrug-resistant pathogens and infectious diseases such as COVID-19 (Yang et al., 2022; Li F. et al., 2023; Li Y. et al., 2023; Khalifa and Al Ramahi, 2024). Their distinctive optical characteristics also support advanced imaging modalities like photoacoustic imaging and surface-enhanced Raman scattering, enabling accurate localization and real-time monitoring of infections. Moreover, AuNP-based nanocarriers can facilitate targeted delivery of therapeutic agents directly to infected sites, thereby minimizing systemic toxicity and enhancing treatment outcomes (Kumar and Chawla, 2021; Wahnou et al., 2023). Continued research is warranted to further investigate their utility in other medical domains, such as cancer therapy, regenerative medicine, biosensing, and immune modulation, ultimately broadening their clinical relevance.

## 10 Conclusion

Through extensive research AuNPs showcase promise as innovative agents against MDR pathogens, demonstrating potent antibacterial properties against a wide range of pathogens. Through this review, we have explored gold nanoparticles’ diagnostic applications, understood their antimicrobial properties, and delved into their unique optical properties that enable sensitive and specific detection of bacterial infections. Combination therapies were also explored displaying efficiency and efficacy, this collaborative approach can aid in overcoming antibiotic resistance and improving current threatened treatment outcomes. Further factors were also covered, including their low toxicity and biocompatibility allowing safe usage when administered in a plethora of therapeutic strategies. Targeted delivery systems such as the functionalizing AuNPs with ligands for specific delivery of antimicrobial agents to infected tissues or cells, have enhanced therapeutic efficacy while minimizing off-target effects. Ongoing research is focused on optimizing the design in addition to the synthesis of AuNPs to enhance their antibacterial activity across multiple medical spectrums. Overall, gold nanoparticles require further research, however, represent a promising class of innovative agents against MDR pathogens, offering a multifaceted approach to combatting antibiotic resistance and improving treatment outcomes in infectious diseases.
